# Medico-Legal Aspect of Skin Grafting

**Published:** 1887-03

**Authors:** 


### MEDICO-LEGAL ASPECT OF SKIN-GRAFTING.

History repeats itself in the presentation now and then of charges for mal-practice against members of the medical profession, which, upon investigation, have proved to be groundless. A unique allegation, in this same spirit of antagonism to physicians, has recently been brought against one of our most pacific practitioners by a charge of assault and battery. It does not appear, however, that it was accompanied with the aggravation of intent to kill, or that any damage was sustained by the party upon whom the assault is claimed to have been made by the physician.

This case was brought to the notice of the public through our daily prints nearly six months ago, but we have refrained from referring to it while under judicial investigation; and now that it has been disposed of by the court, we feel that, as exponents of the enlightened sentiments of the medical profession, we should express our convictions of this unrighteous proceeding.

On August the 30th, 1886, in the presence of Drs. Hardon, Westmoreland and Howell, Dr. Henry Wile, of this city, proposed to a boy of thirteen years to submit to the removal of some small skin-grafts from his arm to be placed upon an extensive ulcerated surface on the head of his cousin, a little girl somewhat younger, whom he had accompanied to the office. The boy readily consented, and minute grafts were excised without causing him any inconvenience.

In the afternoon of the same day, the father of the boy went to the office and charged Dr. Wile with having cut "his son's arm to pieces." He subsequently swore out a warrant charging him with assault and battery; whereupon Dr. Wile waived examination and gave bond in the sum of two hundred dollars for his appearance at the City Court.

The trial was before Judge Van Epps, without a jury, and after reviewing the facts as above given, the Judge stated that the boy had more than ordinary intelligence and discretion, and that a child of his age, under such circumstances of intellectual development, could commit crime and be punished according to law. He considered, therefore, that he had a right to give his consent, so that no crime was committed, and the case was dismissed.

We need not remind our readers that Dr. Wile was clearly justified in his course of treatment, this being a recognized mode of restoration in extensive destruction of the skin.

It is evident that no advantage was taken of the youth, since he acquiesced in the measure and allowed a number of small cuttings to be made without complaint. He was not disfigured in the least, nor was it even alleged that he suffered in any way from the effects of this slight operation. Most medical men have submitted to this; when an occasion called for it, to supply skin-grafts to their own patients, or those of other practitioners, and only ignorance or perversity could lead one to suppose that there was any indignity practiced upon the individual from whom these grafts were taken.

We cannot divest ourselves of the impression that there was something underlying this prosecution which did not spring from paternal regard, and it is a source of regret that in too many instances encouragement is given to such litigation by those who ought to nip such proceedings in the bud by refusing to undertake such cases.

The calm deliberation of Judge Van Epps in weighing all the evidence and law should commend his decision to the judgment of every medical man and every good citizen as a rebuke to all such malicious prosecutions in future, and we congratulate Dr. Wile upon the result in this case.

### ITEMS.

DR. J. B. PENDERGRASS, of Jefferson, Ga., will marry Miss Nellie Egerton, of Louisburg, N. C., on March 3d.

DR. E. H. RICHARDSON, of Cedartown, Vice-president of the Medical Association of Georgia, gave THE JOURNAL a pleasant call recently.

WE had a pleasant call a few days since from Dr. W. A. Strother, of Albany, Ga. Dr. Strother is in the city attending his son, W. A. Strother, jr., a student of the Atlanta Medical College, who has been quite sick with pneumonia.

SCRIBNER'S MAGAZINE for March contains a vigorous and thoughtful paper from the pen of Prof. William James, with the title, "What is an Instinct?" in which he discusses especially the instincts of man, their nature and the laws which govern them.

THE Annual of the Universal Medical Sciences is the title of a new publication to be edited by Dr. Chas. E. Sajous, of Philadelphia. Five volumes of about five hundred pages each will be issued yearly. These will be prepared by something over two hundred editors. Having all the facts relating to a particular subject, each editor will condense these into proper shape for permanent record. The work will be sold for fifteen dollars.

DR. F. H. DARBY, President of the Miami Valley Medical Society, and a graduate of the Ohio Medical College, has had the temerity to refuse to give expert testimony unless paid therefor. The judge promptly sent him to jail. Thence he issued to carry his cause to the Supreme Court of the State. It is to be expected that his professional brethren, who are to reap the benefits of his struggle, will back him with means for conducting the case to a successful issue.—*American Lancet.*

DR. WM. C. WILE, editor of the *New England Medical Monthly*, has been elected to the chair of Nervous Diseases and Electro-therapeutics in the Medico-Chirurgical College of Philadelphia.

WE have received the first number of the *Medical Register*, a new weekly published in Philadelphia by Drs. J. V. Shoemaker and W. C. Wile. It presents a handsome appearance and is well edited. Success to you, gentlemen.

A NEW MEDICAL ASSOCIATION.—The physicians of Paulding, Cobb, Carroll and Douglas counties, in this State, met at Austell a few days since and organized a new medical association to be known as the Sweet Water Medical Association.

TWO DOCTORS JAILED.—On Sunday afternoon, February 27, Drs. I. A. Cofer and R. M. Auten, of this city, both eclectics, were arrested and locked up on the charge of attempting to produce an abortion on a young white woman from Campbell county. On the following day they were carried before a magistrate and required to give a bond of $500 for their appearance at the City Court, which will convene in a few days to hear criminal cases. We forbear comment pending judicial investigation.

THE RUSH MONUMENT.—At the annual meeting of the American Medical Association, held in Washington, D. C., in 1884, a committee was appointed to collect funds for the erection of a suitable monument to the memory of Benjamin Rush, to cost $40,000, the amount to be raised by small subscriptions of one dollar. Dr. A. L. Gihon, of the United States navy, was appointed chairman; Dr. G. H. Rhoe, of Baltimore, secretary, and Dr. J. M. Toner, of Washington, treasurer. It is to be hoped that the profession will respond promptly to this call. Physicians in Georgia can send their contributions to Dr. James A. Gray, secretary of the Medical Association of Georgia, P. O. box 523, Atlanta, and they will be promptly forwarded to the general secretary of the committee.

WOMAN'S MEDICAL COLLEGE.—Cincinnati is to have a new medical college, and this time it is to be an institution exclusively devoted to the medical education of women. A number of our very best teachers are practically engaged in the movement, and success seems to be already assured. One of the old colleges has attempted the plan of having mixed classes, which has proved unsatisfactory to all concerned, and as there seems to be an actual demand for a college where women could receive a medical education, the necessary factors were brought into a harmonious working condition, and we have evolved the Woman's Medical College, of Cincinnati. The innovation has our best wishes, and we predict for the college a brilliant future.—*Lancet.*

THE RELATION OF PHYSICIANS TO THEIR MEDICAL SUPPLIES. The following from the January, 1887, *Ephemeris*, by Dr. Squibb, is, to say the least of it, in bad taste, coming as it does from one who has had as much free advertising from medical journals as he has. Possibly Dr. Squibb has found out that the profession are satisfied with the drugs of other manufacturers, and that this slur at the journals will cause them to "specify Squibb's when prescribing:" "There are a large number of physicians who, for both therapeutics and materia medica, depend largely, if not mainly, upon the traveling salesmen and their pamphlets and lists, and on the advertising pages of the medical journals. The relations of this class to their supplies are most simple and most favorable. They come in very direct and very close contact with the sources of their supplies, and have much less trouble than any other class of artisans. With others smarter, more ingenious and more plausible than they to think for them, and then to apply vigorous mercantile principles to their wants thus suggested for them, they have the least practicable amount of thinking for themselves to do in regard to their remedies and the novelties of the day, and therefore, as they argue, more time to think of and study out their cases. To this class the ready-made prescriptions in the form of beautifully-colored and coated pills, or palatable solutions and mixtures, do not appeal simply as gratifying various degrees of laziness, or indisposition to think for themselves, but they present themselves as true labor-saving devices, skillfully prepared for the over-worked ability to use them, and as giving more time for the higher and more scientific researches of the profession." . . . . .

CASES OF SKIN ERUPTIONS AND SYPHILIS TREATED WITH HORSFORD'S ACID PHOSPHATE.—It appears to me that the "acid phosphate" originally prescribed by Prof. Horsford, of Cambridge, U. S. A., is not so well known in this country as its merits deserve. A glance at the formula will, however, readily convince one of its value in suitable cases. Each fluid drachm gives on analysis 5½ grains of tree phosphoric acid, and nearly four grains of phosphate of lime, magnesia, iron and potash. The following are a few brief notes of some of the cases in which I have prescribed it with complete success.

Mr. G., æt. 69, consulted me November, 1885, for eczema on the arms, legs, palms of the hands and trunk. The patient complained of much debility and nervous exhaustion, and he was a man who had led a very busy business life with much worry. In December, 1885, I prescribed Horsford's acid tonic with much good effect, as in February, 1886, I heard that he was quite well.

Mrs. S., æt. 46, consulted me in December, 1885, for psoriasis, all over the body, more or less, especially on the legs and arms. In January, 1886, I prescribed a teaspoonful of the acid tonic three times a day with marked good effect. Patient had been much exhausted by continuous nursing on an invalid mother.

Mr. C., æt. 64, consulted me in September, 1885, with one of the worst attacks of late syphilis I ever saw. After he had been relieved from the distressing symptoms and ulcerations, I prescribed the acid tonic for epileptiform fits, from which he suffered, with excellent results.

Mr. McJ., æt. 63, consulted me in November, 1885, for lichen ruber, which was accompanied with intolerable itching. He was a nervous, irritable man. I prescribed the acid tonic with the effect that in December he presented himself quite convalescent. —By Mr. James Startin, *Medical Press*, London, England.

MALTINE AS A FOOD-SOLVENT.—Referring to use of maltine in his treatment of indigestion, Dr. Fothergill says: "Then, again, in order to aid the defective action upon starch by the natural diastase being deficient in quantity or impaired in power, we add the artificial diastase, maltine. But, as Dr. Roberts points out, in order to make this ferment operative, it must not be taken after a meal is over. Rather it should be added to the various forms of milk porridge or puddings before they are taken into the mouth. About this there exists no difficulty. Maltine is a molasses-like matter and mixes readily with the milk, gruel, etc., without interfering either with its attractiveness of appearance or its toothsomeness ; indeed, its sweet taste renders the gruel etc., more palatable. A minute or two before the milky mess is placed before the child or invalid, the maltine should be added. If a certain portion of baked flour, no matter in what concrete form, were added to plain milk, and some maltine mixed with it before it is placed on the nursery table, we should hear much less of infantile indigestion and malnutrition."

COPAIBA IN THE TREATMENT OF CROUP.—Dr. P. H. Thompson, Bluffton, Ga., writes: About fifteen years ago, while a student of medicine, my attention was arrested by an article in the *Medical and Surgical Reporter*, advising the use of copaiba in croup. For the last six years I have had very gratifying and unfailing success with all cases of croup, and among the number treated I feel sure that some few at least were cases of that dreadful type called membranous. I have given the balsam in various combinations, sometimes simply emulsionized.

The following mixture, which no doubt can be improved, has been used with pleasing results to myself and patrons:

℞ Bal. copaiba................................f℥i.

Gum acac., pulv..............................℥ss.

Syr. squill...................................f℥i.

Aqua menth, q. s.............................f℥iv.

M.

Sig.—Dose, one teaspoonful every 2 to 6 hours.

PRACTICABILITY OF ESTABLISHING A FISTULOUS OPENING IN THE HUMAN SUBJECT FROM THE GALL BLADDER INTO THE DUODENUM.—The *British Medical Journal*, of February 5th, contains the paper of Dr. J. McF. Gaston, with the above title, presented at the meeting of the British Medical Association in Brighton. It is illustrated by ten cuts from original drawings. It is an interesting feature of the investigations of Dr. Gaston that his original experiments, published in THE JOURNAL, should have been confirmed by those of Golzi without having any knowledge of the prior results. The republication of Dr. Gaston's article on this subject from the Reference Hand-book of the Medical Sciences by the *Journal de Medicine*, of Paris, recently, must bring his work to the knowledge of the profession in Europe, and will doubtless lead to the application of this operation to the relief of obstructions of the common bile duct in the human subject at an early day.

CATARRHAL PHTHISIS.—T. J. Hargan, M. D., Asheville, N. C., writes: On the 31st of July, 1886, I was consulted by Mr. S., of Tennessee. Patient 27 years of age; weight, 130 pounds; respiration, 28 per minute; pulse, 110 and temperature 101.5. From the subjective examination I learned that his family history was good, and that previous to two years ago he had always had good health. About that time he "took a severe cold in the head," the results of which have continued to this day. He has putrid discharges from his nose, tonsilitis and pharyngitis; severe pains over his eyes, especially in the centre of the forehead; has almost constant cough; expectorates freely; has had severe hemorrhages from the lungs within the past year; has suffered from recurrent attacks of bilious fever about every four weeks. Physical examination revealed considerable dullness posteriorly immediately below the right scapula; some dullness below the right clavicle; some bronchitis, but not extensive. On making nasal examination, found the nares dry and scaly near the external orifice. The mucous membrane lining of the nasal cavity was much inflamed and thickened. The velum palati was elongated, inflamed and much swollen. The nasal septum was perforated near the middle, a probe readily passing through from one side to the other. The discharge from the nose was dark green and of a very offensive odor. The pharynx and larynx were much inflamed, the catarrhal condition having extended downward. The eustachian tubes were also much affected, indicated by a constant roaring in the patient's ears, and his hearing was much impaired. The disease had also extended to the frontal sinus, as shown by the constant pain in the forehead; this frontal pain he had not been free from for a year. The lachrymal ducts were also involved, being occasionally closed so the tears flowed out over the cheeks.

The patient was placed under the following treatment: Oxygen and nitrous oxide, each one part; common air, two parts. Of this mixture he inhaled five gallons twice daily.

He was also treated with a spray consisting of aqueous solution of bicarbonate of soda and chloride of sodium. This spray was thrown in the nose and throat once daily until the parts were thoroughly cleansed. Twice daily, by means of the Sass spray apparatus, the following was vaporized and inhaled in nose and mouth:

| | | |
|---|---|---|
| ℞ Ol. | Eucalyptol | gtt. v. |
| " | Gaultheria | " ii. |
| " | Tar | " xx. |
| | Vaseline | ℥i. |

M.

Of this one drachm was vaporized and inhaled at a sitting, the patient inhaling it through the mouth and nose.

All the symptoms were rapidly overcome, the frontal headache leaving entirely within the first week of treatment. The above was continued for thirty days, when he had improved so rapidly that the treatment was suspended. He gained 16 pounds in weight in 16 days. He was furnished, at his own request, with a Sass spray apparatus, which he continued to use at home, and he now writes me that he is entirely well.

A case of chloroform death occurred in Philadelphia two weeks ago in the practice of Dr. Wm. H. Pancoast. The anæsthetic was administered to a man aged thirty for the purpose of having adhesions of the finger tendons of the hand broken up. The usual remedies were applied without avail. The autopsy revealed the kidneys much diseased and an unusually fatty liver and heart, and a thin right ventricle.—*Maryland Med. Journal.*

